# Anti‐Arthritic Potential of Pyrazoline Derivative Against Complete Freund's Adjuvant Induced Arthritis in Rats

**DOI:** 10.1111/jcmm.71030

**Published:** 2026-02-24

**Authors:** Jian Li, Irfan Anjum, Halima Qadir, Faiza Naseer, Mehreen Arif, Muhammad Riaz, Rabia Gul, Madiha Kanwal, Bei Zhuang

**Affiliations:** ^1^ Department of Joint and Sports Medicine The Fourth People's Hospital of Jinan Jinan China; ^2^ Department of Basic Medical Sciences, Shifa College of Pharmaceutical Sciences Shifa Tameer‐e‐Millat University Islamabad Pakistan; ^3^ Department of Biosciences Shifa Tameer‐e‐Millat University Islamabad Pakistan; ^4^ Quaid‐e‐Azam University Islamabad Pakistan; ^5^ Deparmatment of Pharmaceutical Chemistry, Riphah Institute of Pharmaceutical Sciences Riphah International University Islamabad Pakistan; ^6^ Department of Orthopedics PLA Naval Medical Center Shanghai China

**Keywords:** arthritis, complete Freund's adjuvant (CFA), paw edema, proinflammatory cytokines, pyrazoline derivatives

## Abstract

This research explored the potential of a synthesised pyrazoline derivative 5‐ethoxy 5‐hydroxy 3‐methyls 4, 5‐dihydro 1Hpyrazol 1 yl (pyridine 4 yl) methanone [5‐E‐5‐H‐PD], against arthritis using a Complete Freund's Adjuvant (CFA)‐induced arthritis in a rat model. Sprague–Dawley rats were used to induce arthritis via subplantar injection of CFA (0.1 mL) into their right hind paw. Animals were divided into 6 groups (*n* = 4): normal, arthritis, standard drug (methotrexate 1 mg/kg intraperitoneally), and 3 treatment groups receiving 5‐E‐5‐H‐PD, 10, 20 and 40 mg/kg orally for 21 days. Clinical signs (paw volume and arthritis score), pro‐inflammatory cytokines, and histopathological alterations were evaluated. The 5‐E‐5‐H‐PD groups showed a reduction in paw edema in a dose‐dependent manner. On day 21, paw volume in the 40 mg/kg dose animals decreased significantly to 2.31 ± 0.12 mm compared to 4.82 ± 0.14 mm in the disease animals (*p* < 0.001). Arthritis scores reduced from 3.8 ± 0.2 (control) to 1.5 ± 0.3 in the high‐dose treatment group. Serum IL‐10, TNF‐α, and NF‐κB levels were significantly reduced to 66.75 ± 3.0 pg/mL, 34.50 ± 1.8 pg/mL and 9.50 ± 0.6 pg/mL respectively, compared to the arthritis induced rats 129.8 ± 2.0 pg/mL, 77.75 ± 1.5 pg/mL and 28.50 ± 1.3 pg/mL respectively, compared to the arthritis induced rats (112.3 ± 5.5, 96.8 ± 4.3, 123.1 ± 6.2 pg/mL, *p* < 0.001). Histopathology analysis confirmed reduced synovial hyperplasia and inflammatory infiltration in treated joints. The pyrazoline derivative, 5‐E‐5‐H‐PD, demonstrated significant anti‐arthritic effects in the CFA‐induced rat model by reducing inflammation, cytokine expression and joint destruction. These findings support further investigation into pyrazoline‐based compounds as promising therapeutic agents for RA.

## 1 Introduction

Rheumatoid arthritis is characterised by inflammation of synovial joints, cartilage degradation, bone erosion and joint deformities because it's a chronic and progressive autoimmune disease. It affects approximately 0.1%–2% of the global population, imposing a substantial burden on quality of life [1]. Pathophysiologically, rheumatoid arthritis (RA) involves an imbalance between oxidative stress and antioxidant defences within the synovial environment. Reactive oxygen species (ROS), generated during inflammation, cause activation of NF‐κB, which in turn promotes the transcription of TNF‐α and other pro‐inflammatory cytokines [2, 3]. This cascade culminates in synovitis and cartilage destruction, largely driven by interleukins such as IL‐1β and IL‐6. Moreover, TNF‐α contributes to osteoclastogenesis by activating the receptor activator of NF‐κB ligand and downregulating the osteoprotegerin, thereby exacerbating joint damage [4]. Experimental animal models using Complete Freund's Adjuvant (CFA) or formaldehyde are broadly accepted to mimic the clinical and pathological features of human RA [5].

Current pharmacological management includes nonsteroidal anti‐inflammatory drugs (NSAIDs) such as diclofenac, ibuprofen, piroxicam and disease‐modifying anti‐rheumatic drugs (DMARDs), i.e., methotrexate. While these agents alleviate symptoms and slow disease progression, their use for a longer period is associated with a lot of adverse effects, including an increase in gastric juice leading to gastrointestinal ulceration, cardiovascular complications and hepatic or renal toxicity [1, 6]. Hence, the search for safer and more effective alternatives is ongoing, with increased interest in synthetic heterocyclic compounds. Pyrazoles are five‐membered heterocyclic rings with two nitrogen atoms, exhibiting a wide array of pharmacological activities. Their therapeutic activities include potent action against pathogenic microorganisms, treatment of inflammation and joint pain, and cancer as well [7, 8]. Their derivatives, including pyrazoline‐3‐ones and pyrazolidine‐3,5‐diones, have historically demonstrated efficacy in pain and inflammation management. Compounds such as antipyrine, phenazone and sulfinpyrazone have been employed as antipyretics, anti‐inflammatories and uricosuric agents [9, 10].

**FIGURE 1:**
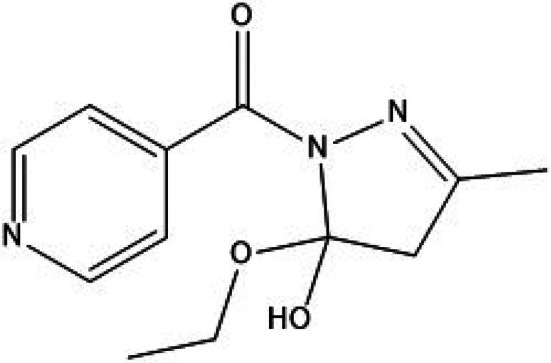
5‐ethoxy‐5‐hydroxy‐3‐methyl‐4,5‐dihydro‐1H‐pyrazol‐1‐yl(pyridin‐4‐yl)methanone (5‐E‐5‐H‐PD).

Pyrazoline derivatives represent a well‐established class of heterocyclic compounds widely explored for their diverse pharmacological potential, including anti‐inflammatory, analgesic, anticancer, antimicrobial and antioxidant activities [11]. The test compound used in the present study, 5‐ethoxy‐5‐hydroxy‐3‐methyl‐4,5‐dihydro‐1H‐pyrazol‐1‐yl(pyridin‐4‐yl)methanone (5‐E‐5‐H‐PD), is a synthetically designed pyrazoline scaffold bearing ethoxy, hydroxy and pyridinyl moieties that are known to enhance biological activity [12]. This compound was synthesised through the conventional cyclization of substituted chalcones with hydrazine hydrate, yielding a dihydro‐pyrazoline ring with multifunctional substituents positioned to modulate both lipophilicity and hydrogen‐bonding capacity [13]. Its molecular framework contains a pyridine ring, which often improves anti‐inflammatory potential through enhanced receptor binding and metal‐chelation effects, while the presence of hydroxyl and alkoxy (ethoxy) groups increases radical‐scavenging ability and contributes to improved solubility. Literature reports show that structurally similar pyrazoline analogs exhibit inhibition of key inflammatory mediators such as COX‐2, TNF‐α and IL‐6, suggesting a mechanistic rationale for evaluating 5‐E‐5‐H‐PD in rheumatoid arthritis models [14, 15]. From a SAR perspective, electron‐donating substitutes (−OH, −OEt) at the 5‐position and a pyridinyl carbonyl group at the N‐1 position often enhance anti‐inflammatory and antioxidant activity by stabilising the pyrazoline ring and facilitating interaction with inflammatory targets, as shown in Figure 1. Therefore, the chemical architecture of 5‐E‐5‐H‐PD indicates strong potential as a multi‐target therapeutic candidate, supporting its evaluation in the CFA‐induced arthritis model.

In this context, the current work explores the pharmacological potential of a novel pyrazoline derivative, (5‐E‐5‐H‐PD), in mitigating RA‐like symptoms in a CFA‐induced murine arthritis model. This work aims to provide preclinical evidence of its efficacy using behavioural, biochemical, histological and molecular docking approaches.

## 2 Methods

### 2.1 Animals

The ethical approval for the conduction of the study was granted by the Animal Ethics Committee of Shifa College of Pharmaceutical Sciences, Shifa Tameer e Millat University (SCPS‐STMU/Animal Ethics Approval/IRB # 041‐24). For the investigation, 150–200 g weight of healthy, 8–10 weeks old, male or female Sprague–Dawley rats were kept in standard laboratory conditions (27°C, 35%–50% humidity). All the polypropylene cages (four rats/cage) were subjected to a 12‐h light/12‐h dark cycle. They were provided a standard pellet diet and unlimited access to water. All the animals were acclimatised for a week before the experiment.

### 2.2 Methodology

Randomly, twenty‐four rats were divided into six groups (*n* = 4/group).

Group I: Vehicle: Normal saline, no CFA.

Group II: Disease, Arthritis received only the CFA 0.1 mL injection.

Group III: Standard drug methotrexate 1 mg/kg intraperitoneally as a reference.

Groups IV, V and VI: Treatment groups received 10, 20 and 40 mg/kg of pyrazoline derivative, 5‐E‐5‐H‐PD, respectively, on alternate days starting from day 7 post CFA‐induction to day 28.

Animals were sacrificed on the 28th Day to conduct histopathological and biochemical evaluation [16].

### 2.3 Induction of Arthritis

Every group of rats had its right hind leg sterilised with 70% alcohol, and each animal received 0.1 mL of CFA containing 10 mg/mL of heat‐killed *Mycobacterium tuberculosis* subplantarly in the hind paw under light diethyl ether anaesthesia, except for Vehicle control group 1, which received normal saline [17].

### 2.4 Assessment of Arthritis

#### 2.4.1 CFA‐Induced Arthritic Paw Edema

Using a Vernier calliper, the thickness of both hind paws was measured on 0, 7, 14, 21 and 28 days following CFA injection. The results were statistically compared among groups to evaluate the anti‐inflammatory effect of the test compound [17].

#### 2.4.2 Arthritis Scoring Index

The progression and intensity of diseases were assessed using a visual scoring system of the clinical examination on a scale of 0–4 per limb.

No physical change: score 0.

Slight swelling and erythema of the limb: score 1.

Moderate swelling and redness: scores 2.

Gross swelling on the limb and erythema on joints: score 3.

Deformity and unable to move the limb: score 4.

A score greater than 1 indicates the induction of disease when examining the hind leg. An eight is the maximum score for arthritis. Beginning on days 0 and 7 following the CFA injection, the measurement was repeated on days 14, 21 and 28 post‐CFA injection [16, 17].

### 2.5 Haematology Profile

On the 28th day of post‐CFA injection, blood samples were collected through cardiac puncture after anaesthesia. These blood collection tubes were stored at 4°C. Haematological parameters were measured, including C‐reactive protein (CRP) and rheumatoid factor (RF). These included haemoglobin content (HGB), total red blood cell count (RBC), erythrocyte sedimentation rate (ESR) and total leukocyte count (TLC). Liver enzyme levels, like Aspartate aminotransferase (AST), Alkaline phosphatase (ALP) and Alkaline transferase (ALT), were analysed using standard diagnostic kits [16, 17].

### 2.6 In Vivo Antioxidant Assays

Serum antioxidant levels were evaluated for Glutathione (GSH), Catalase (CAT), Superoxide dismutase (SOD) and Malondialdehyde (MDA). Following the manufacturer's instructions, mouse ELISA kits were used to measure these antioxidant enzymes in the serum [17].

### 2.7 Proinflammatory Biomarkers

The blood samples were allowed to clot for half an hour and centrifuged for 10 min at 3000 rpm to separate the serum. It was used to quantify the proinflammatory biomarkers, including TNF‐α, NF‐κB and IL‐10, using ELISA reagent kits following the manufacturer's instructions. Serum was stored at −20°C until further examination [18].

### 2.8 Histological Assessment of Ankle Joints

Rat ankle joints were harvested, fixed in 10% formalin, and decalcified using an EDTA‐based solution. These tissues were embedded in paraffin, sectioned at 5 μm‐thickness, stained with haematoxylin and eosin (H & E), and examined under a light microscope to assess the synovial hyperplasia, pannus formation and joint deformity [17].

### 2.9 Molecular Docking Analysis

The 3‐D structures of target proteins IL‐10 (PDB: 3LQM), TNF‐alpha (PDB: 2AZ5) and NF‐κB (PDB: 1SVC) were retrieved from the RCSB protein data bank. The ligand (5‐E‐5‐H‐PD) structures were obtained from PubChem (accessed August 11, 2024, via https://pubchem.ncbi.nlm.nih.gov/). All the missing atoms and valencies were corrected in the PDB molecule after removing all those water molecules from the 3D structure that did not participate in the ligand interaction. The atomic coordinates were generated after the conversion of the file into PDBQT by using PyRx (A free version for molecular docking was available). These PDB coordinates of the target protein and 6‐Methoxyflavone were optimised for ligand‐receptor docking by the software Drug Discovery Studio version 3.0, and the missing residues were added. During optimization, these coordinates were stable with the confirmation of the least energy. After that, Biovia Drug Discovery Studio version 3.0 was used to examine the active binding sites of target proteins. These active sites are the ligand's coordinates in the initial target protein grids (https://discover.3ds.com/discovery‐studio‐visualizer). The virtual screening was utilised to study the binding affinities of ligands and proteins, pharmacological activities at specific sites, and enzyme and protein interactions. All default docking techniques were employed using PyRx; the coordinates were all established in the grids and put in the active site pocket centre, and for stable, significant interactions, minimum binding energies were the most appropriate [19, 20].

## 3 Results

### 3.1 Effect of 5‐E‐5‐H‐PD on CFA‐Induced Paw Edema

**FIGURE 2:**
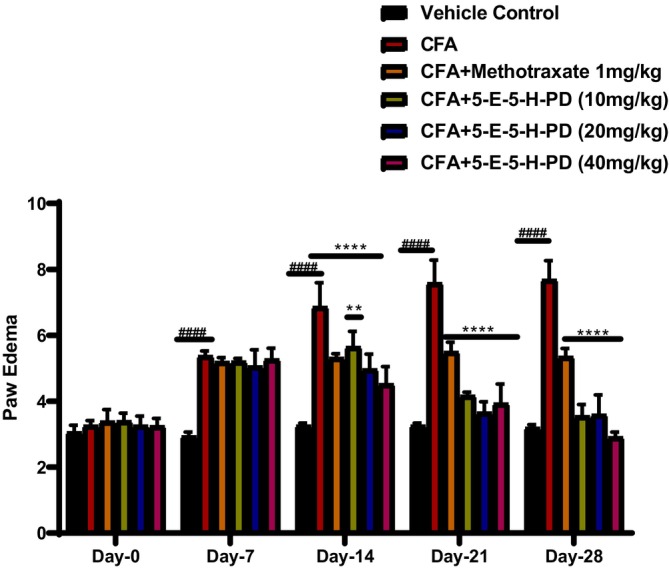
Effect of 5‐E‐5‐H‐PD on CFA‐induced paw edema. ^####^ *p* < 0.00001 compared to the vehicle control group, **p < 0.001 compared to CFA, ****p < 0.00001 compared to CFA. Two‐way ANOVA followed by post hoc Dunnett's test for comparison between groups.

CFA treatment has noticeably induced paw edema compared to the control animals (Figure 2). This augmentation in paw edema was significantly reduced by methotrexate as well as the test compound in a graded manner throughout treatment.

At the detection time points, CFA treatment significantly induced paw edema compared to the normal animals. The augmentation in paw edema was observed on days 7, 14, 21 and 28, with the most pronounced effect seen on day 28. However, this increase in paw edema was progressively reduced by methotrexate and the pyrazoline derivative (5‐E‐5‐H‐PD) throughout the treatment period. The highest dose of 5‐E‐5‐H‐PD (40 mg/kg) demonstrated the most substantial reduction in paw edema, suggesting its effective anti‐inflammatory properties. The statistical significance was noted for CFA‐induced edema and the reduction observed with 5‐E‐5‐H‐PD treatment.

### 3.2 Effect of 5‐E‐5‐H‐PD on Arthritic Score

**FIGURE 3:**
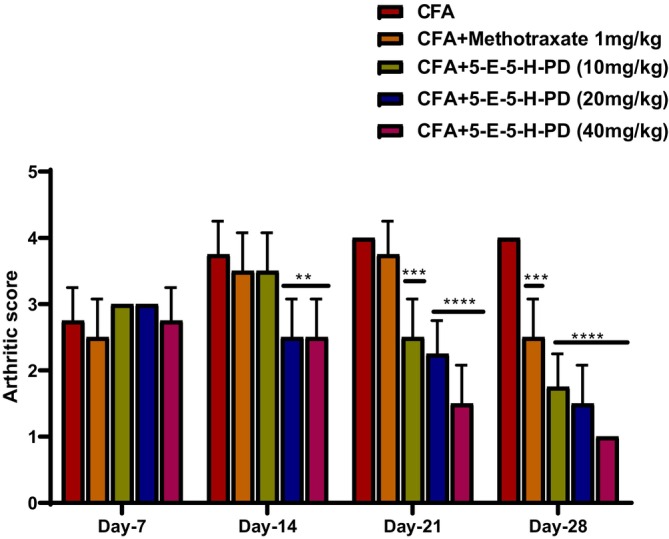
Effect of 5‐E‐5‐H‐PD on CFA‐induced arthritic score. ***p < 0.0001 compared to the vehicle control group, **p < 0.001 compared to CFA, ****p < 0.00001 compared to CFA. Two‐way ANOVA followed by post hoc Dunnett's test for comparison between groups.

CFA CFA‐induced group has shown a marked increase in arthritic score over time. This rise in arthritic score was reduced in a graded manner by the test compound, with the most significant result at 28 days, as shown in Figure 3.

**FIGURE 4:**
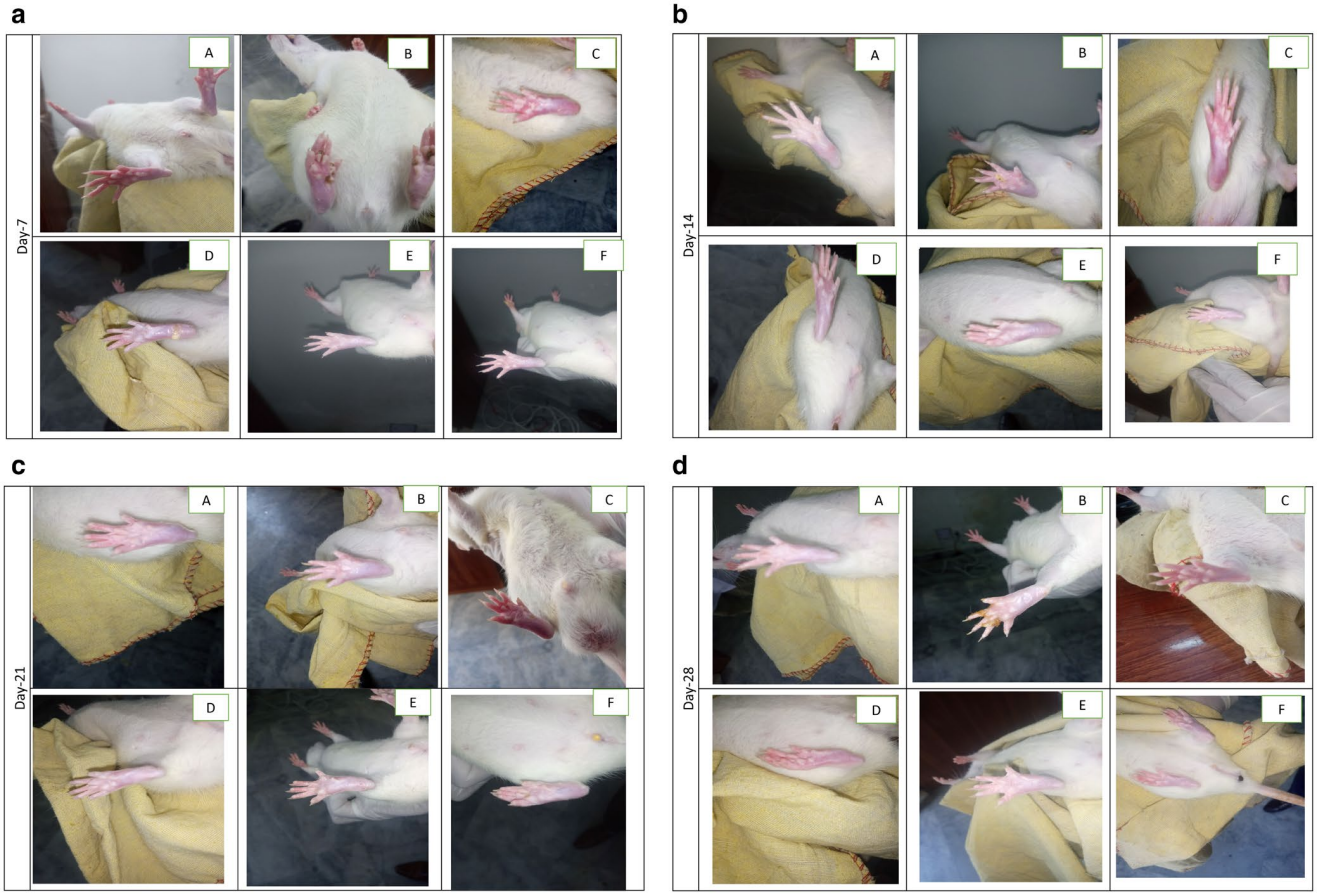
(a) At Day 7, (A) Vehicle, (B) Disease (CFA), (C) Standard (CFA + methotrexate 1 mg/kg), (D) CFA + (5‐E‐5‐H‐PD) 10 mg/kg, (E) CFA + (5‐E‐5‐H‐PD) 20 mg/kg, (F) CFA+(5‐E‐5‐H‐PD) 40 mg/kg. (b) At Day 14, (A) Vehicle, (B) Disease (CFA), (C) Standard (CFA + methotrexate 1 mg/kg), (D) CFA + (5‐E‐5‐H‐PD) 10 mg/kg, (E) CFA+(5‐E‐5‐H‐PD) 20 mg/kg, (F) CFA + (5‐E‐5‐H‐PD) 40 mg/kg. (c) At Day 21, (A) Vehicle, (B) Disease (CFA), (C) Standard (CFA + methotrexate 1 mg/kg), (D) CFA+(5‐E‐5‐H‐PD) 10 mg/kg, (E) CFA + (5‐E‐5‐H‐PD) 20 mg/kg, (F) CFA + (5‐E‐5‐H‐PD) 40 mg/kg. (d) At Day 28, (A) Vehicle, (B) Disease (CFA), (C) Standard (CFA + methotrexate 1 mg/kg), (D) CFA + (5‐E‐5‐H‐PD) 10 mg/kg, (E) CFA + (5‐E‐5‐H‐PD) 20 mg/kg, (F) CFA + (5‐E‐5‐H‐PD) 40 mg/kg.

The CFA‐induced group showed a remarkable increase in arthritic score compared to the normal animals because the arthritic score was negligible in normal animals. Treatment with methotrexate and 5‐E‐5‐H‐PD reduced this increase in a dose‐dependent manner (Figure 4a–d). The most statistically significant reduction was seen at the highest dose of 40 mg/kg of 5‐E‐5‐H‐PD as compared to CFA.

### 3.3 5‐E‐5‐H‐PD Effects on Serum Biochemical Parameters

The significantly elevated serum levels of AST, ALT, CRP, anti‐CCP and RF in the CFA group were observed compared to control animals. However, the administration of (5‐E‐5‐H‐PD) to the treated group showed a decline in all the above‐mentioned parameters as compared with the CFA CFA‐induced group (Table 1).

**TABLE 1:** Shows the 5‐E‐5‐H‐PD effects on serum biochemical parameters: Rheumatic factor (RF), C‐reactive protein (CRP), anti‐citrullinated protein (Anti‐CCP), aspartate aminotransferase (AST) and alanine transaminase (ALT).

Test	Vehicle control	CFA	CFA + methotrexate 1 mg/kg	CFA + 5‐E‐5‐H‐PD (10 mg/kg)	CFA + 5‐E‐5‐H‐PD (20 mg/kg)	CFA + 5‐E‐5‐H‐PD (40 mg/kg)
RF IU/mL	5.35 ± 0.2	54.73 ± 1.6^####^	10.93 ± 0.5****	33 ± 1.4****	24.25 ± 0.6****	13.05 ± 1.1****
CRP mg/L	1.20 ± 0.2	37.03 ± 1.9^####^	21.45 ± 1.0****	28.05 ± 1.6**	24.60 ± 1.3****	37.7 ± 0.3****
Anti‐CCP U/mL	0.70 ± 0.1	63.2 ± 1.9^####^	34.6 ± 1.7****	44.1 ± 0.7****	35.1 ± 1.8****	35.6 ± 0.2****
AST U/L	53.30 ± 1.3	217.3 ± 2.2^####^	59.75 ± 1.4****	102.1 ± 0.6****	80.25 ± 2.0****	46.75 ± 1.7****
ALT U/L	34.25 ± 1.4	186.3 ± 1.7^####^	66.00 ± 2.1****	80.75 ± 2.2****	72.75 ± 1.4****	50.25 ± 2.1****

*Note:* Values are expressed as mean ± SEM (*n* = 6).

^####^ *p* < 0.0001 vs. vehicle control.

** *p* < 0.01.

**** *p* < 0.0001 vs. CFA group.

### 3.4 5‐E‐5‐H‐PD Effects on Haematological Parameters

The hematologic changes were noted in those rats in which CFA injection was administered. A significant decline was noted in haemoglobin (Hb), red blood cell (RBC) counts, and an upsurge in erythrocytic sedimentation rate (ESR) and total leukocyte count (TLC). Treatment groups have shown significantly improved changes (Table 2).

**TABLE 2:** Shows the 5‐E‐5‐H‐PD effects on haematological parameters: RBC count, Hb, ESR and TLC.

Test	Vehicle control	CFA	CFA + methotrexate 1 mg/kg	CFA + 5‐E‐5‐H‐PD (10 mg/kg)	CFA + 5‐E‐5‐H‐PD (20 mg/kg)	CFA + 5‐E‐5‐H‐PD (40 mg/kg)
RBC × 10^6^ μ/L	6.02 ± 0.8	2.92 ± 0.2^####^	4.97 ± 0.3****	4.92 ± 0.6****	5.77 ± 0.1****	6.02 ± 0.5****
Hb g/dL	10.85 ± 0.2	7.27 ± 0.5^###^	11.55 ± 0.4****	9.82 ± 0.2**	11.63 ± 0.4****	12.28 ± 0.2****
ESR mm/h	8.12 ± 0.2	102.5 ± 2.9^####^	24.75 ± 1.5****	41.00 ± 1.2****	33.75 ± 1.7****	14.25 ± 0.8****
TLC × 10^9^/L	4.62 ± 0.1	18.35 ± 1.4^####^	5.70 ± 0.2****	4.27 ± 0.1****	2.67 ± 0.4****	2.30 ± 0.1****

*Note:* Values are expressed as mean ± SEM (*n* = 6).

^###^ *p* < 0.001.

^####^ *p* < 0.0001 vs. vehicle control.

** *p* < 0.01.

**** *p* < 0.0001 vs. CFA group.

### 3.5 5‐E‐5‐H‐PD Effects on Oxidative Stress Markers

The alteration in oxidative stress markers like MDA, GSH, CAT and SOD was observed in the serum of all animals. Results showed that MDA levels were significantly increased in the CFA‐induced disease group, while GSH, CAT and SOD levels were decreased in these animals as compared to the control animals. The serum collected from 5‐E‐5‐H‐PD‐treated animals showed a decline in MDA level and an increase in other parameters compared to the arthritis induced by the CFA group, as shown in Table 3.

**TABLE 3:** Shows the 5‐E‐5‐H‐PD effects on oxidative stress markers: Superoxide dismutase (SOD), catalase (CAT), glutathione (GSH) and malondialdehyde (MDA).

Test	Vehicle control	CFA	CFA + methotrexate 1 mg/kg	CFA + 5‐E‐5‐H‐PD (10 mg/kg)	CFA + 5‐E‐5‐H‐PD (20 mg/kg)	CFA + 5‐E‐5‐H‐PD (40 mg/kg)
SOD U/mg	21.00 ± 0.9	8.50 ± 0.6^####^	16.25 ± 0.9****	7.75 ± 0.4	16.75 ± 0.8****	18.25 ± 0.8****
CAT U/mg	29.25 ± 1.6	10.75 ± 0.8^####^	15.00 ± 1.4	11.75 ± 0.8	21.00 ± 0.9***	25.50 ± 2.1****
GSH U/mg	54.25 ± 1.6	24.75 ± 1.4^####^	36.00 ± 1.4**	32.00 ± 1.0*	34.00 ± 1.4**	47.25 ± 1.7****
MDA µM	9.75 ± 0.8	30.00 ± 1.0^####^	17.00 ± 1.0****	25.25 ± 2.0	16.00 ± 2.0****	9.50 ± 0.6****

*Note:* Values are expressed as mean ± SEM (*n* = 6).

^####^ *p* < 0.0001 vs. vehicle control.

* *p* < 0.05.

** *p* < 0.01.

*** *p* < 0.001.

**** *p* < 0.0001 vs. CFA group.

### 3.6 5‐E‐5‐H‐PD Effects on Proinflammatory Cytokines

The alteration of proinflammatory cytokines like IL‐10, TNF‐α and NF‐κB in the serum collected from all animals was checked. A significant upsurge in these biomarkers was observed in the arthritic animals induced by CFA compared to the control animals. The serum collected from the blood of animals treated with 5‐E‐5‐H‐PD showed a statistically significant decline compared to the disease group (Table 4).

**TABLE 4:** Shows the 5‐E‐5‐H‐PD effects on proinflammatory cytokines: Interleukin‐10 (IL‐10), tumour necrosis factor‐alpha (TNF‐α) and nuclear factor kappa‐B (NF‐κB).

Test	Vehicle control	CFA	CFA + methotrexate 1 mg/kg	CFA + 5‐E‐5‐H‐PD (10 mg/kg)	CFA + 5‐E‐5‐H‐PD (20 mg/kg)	CFA + 5‐E‐5‐H‐PD (40 mg/kg)
IL‐10 Pg/mL	63.25 ± 2.3	129.8 ± 2.0^####^	102.3 ± 3.2***	105.5 ± 4.7***	101.3 ± 3.0****	66.75 ± 3.0****
TNF‐α Pg/mL	39.50 ± 2.9	77.75 ± 1.5^####^	48.50 ± 1.7****	62.00 ± 1.5***	47.50 ± 2.5****	34.50 ± 1.8****
NF‐κB Pg/mL	11.75 ± 1.2	28.50 ± 1.3^####^	15.00 ± 1.5****	18.25 ± 1.6***	16.25 ± 1.2****	9.50 ± 0.6****

*Note:* Values are expressed as mean ± SEM (*n* = 6).

^####^ *p* < 0.0001 vs. vehicle control.

*** *p* < 0.001.

**** *p* < 0.0001 vs. CFA group.

### 3.7 Histological Assessment of Ankle Joints

**FIGURE 5:**
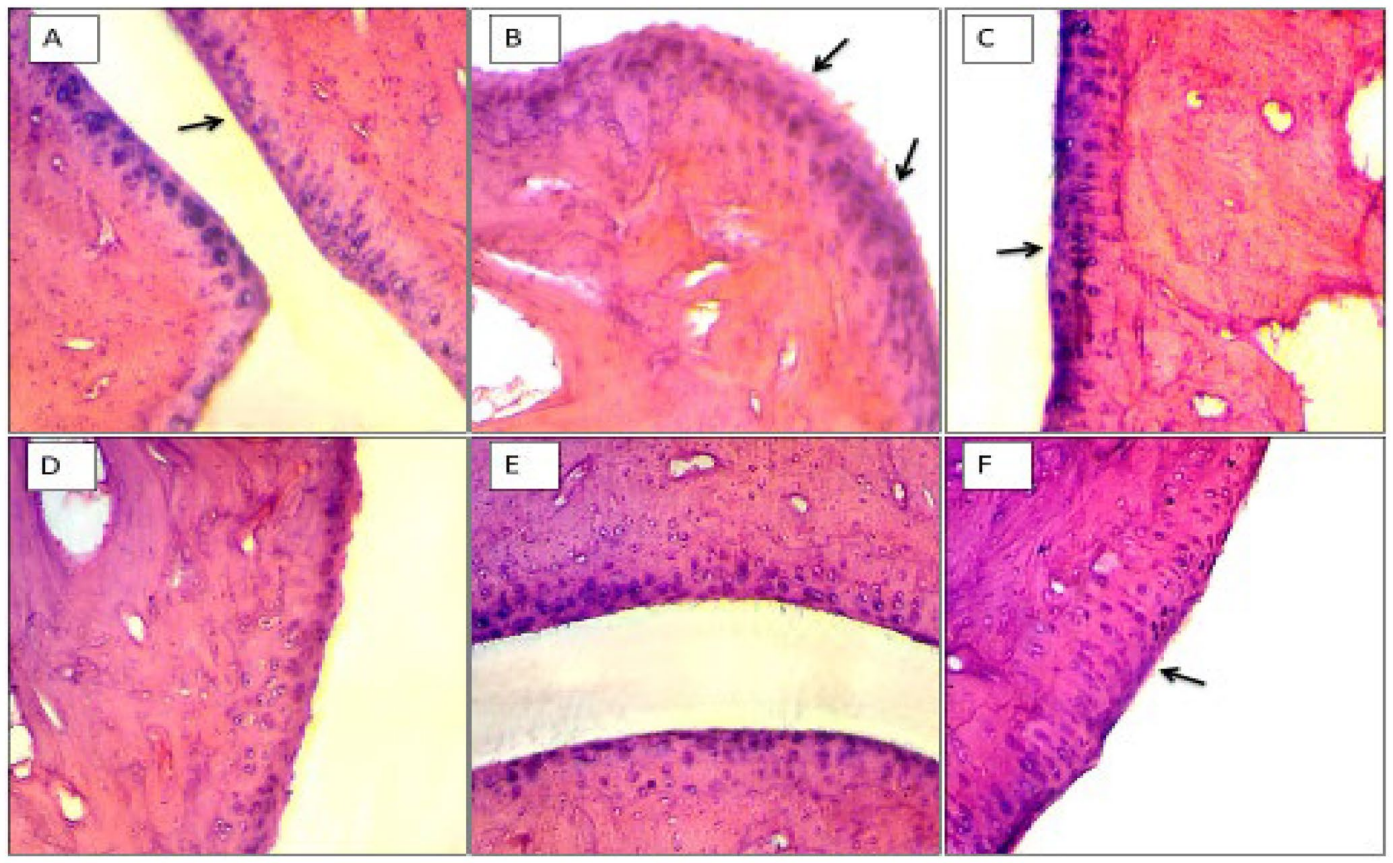
Histological analysis of arthritic joints. Haematoxylin and eosin staining of the knee joint cartilage tissues in the different treatment groups. (A) The control group shows a preserved morphological structure with no signs of cartilage degradation. (B) The CFA group shows chondrocytes that are unable to maintain their repair activity, with subsequent loss of the cartilage tissue. (C–F) Treated groups. Magnification: 20×.

Histological analysis of ankle joints has shown significant deformity in CFA CFA‐induced group, as shown in Figure 5B compared to the normal saline Figure 5A. This deformity was significantly improved by methotrexate (Figure 5C) and treated groups 10 mg/kg (Figure 5D) and 20 mg/kg (Figure 5E). However, normal presentation was seen in the group treated with the highest dose of 5‐E‐5‐H‐PD (Figure 5F).

### 3.8 Molecular Docking of 5‐E‐5‐H‐PD With the Inflammatory Mediators Interleukin‐10 (IL‐10), Tumour Necrosis Factor‐Alpha (TNF‐α), Nuclear Factor Kappa‐B (NF‐κB)

Molecular docking results have shown that 5‐E‐5‐H‐PD has significant binding with inflammatory mediators. Table 5A shows the −5.9 binding energy of IL‐10, Table 5B shows the −6.2 binding energy of TNF‐α, and Table 5C shows the −5.6 binding energy of NF‐κB. So, the lowest binding energy of 5‐E‐5‐H‐PD was reported with TNF‐α.

**TABLE 5:** Binding energy of (A) IL‐10, (B) TNF‐α and (C) NF‐κB.

Ligand	Binding affinity	rmsd/ub	rmsd/lb
**(A)**			
3lqm‐IL‐10_169488867_uff_E = 402.19	−5.9	0	0
3lqm‐IL‐10_169488867_uff_E = 402.19	−5.6	3.188	2.118
3lqm‐IL‐10_169488867_uff_E = 402.19	−5.6	3.44	2.057
3lqm‐IL‐10_169488867_uff_E = 402.19	−5.2	14.415	12.053
3lqm‐IL‐10_169488867_uff_E = 402.19	−5	13.265	11.783
3lqm‐IL‐10_169488867_uff_E = 402.19	−5	14.212	13.186
3lqm‐IL‐10_169488867_uff_E = 402.19	−5	13.742	11.974
3lqm‐IL‐10_169488867_uff_E = 402.19	−5		41.71
3lqm‐IL‐10_169488867_uff_E = 402.19	−4.9	5.308	3.675
**(B)**			
2az5‐TNF_169488867_uff_E = 402.19	−6.2	0	0
2az5‐TNF_169488867_uff_E = 402.19	−6.2	20.976	19.504
2az5‐TNF_169488867_uff_E = 402.19	−6.2	20.83	19.387
2az5‐TNF_169488867_uff_E = 402.19	−6.2	5.823	2.141
2az5‐TNF_169488867_uff_E = 402.19	−6.1	4.181	2.437
2az5‐TNF_169488867_uff_E = 402.19	−6.1	3.382	1.935
2az5‐TNF_169488867_uff_E = 402.19	−6	5.489	2.516
2az5‐TNF_169488867_uff_E = 402.19	−6	3.883	2.545
2az5‐TNF_169488867_uff_E = 402.19	−5.9	5.89	2.992
**(C)**			
1svc‐NF_169488867_uff_E = 402.19	−5.9	0	0
1svc‐NF_169488867_uff_E = 402.19	−5.6	14.321	13.25
1svc‐NF_169488867_uff_E = 402.19	−5.5	2.292	1.775
1svc‐NF_169488867_uff_E = 402.19	−5.4	16.199	15.118
1svc‐NF_169488867_uff_E = 402.19	−5.4	34.966	33.451
1svc‐NF_169488867_uff_E = 402.19	−5.1	30.798	29.768
1svc‐NF_169488867_uff_E = 402.19	−5		2.09
1svc‐NF_169488867_uff_E = 402.19	−5	31.245	29.129
1svc‐NF_169488867_uff_E = 402.19	−4.9	30.262	29.137

**FIGURE 6:**
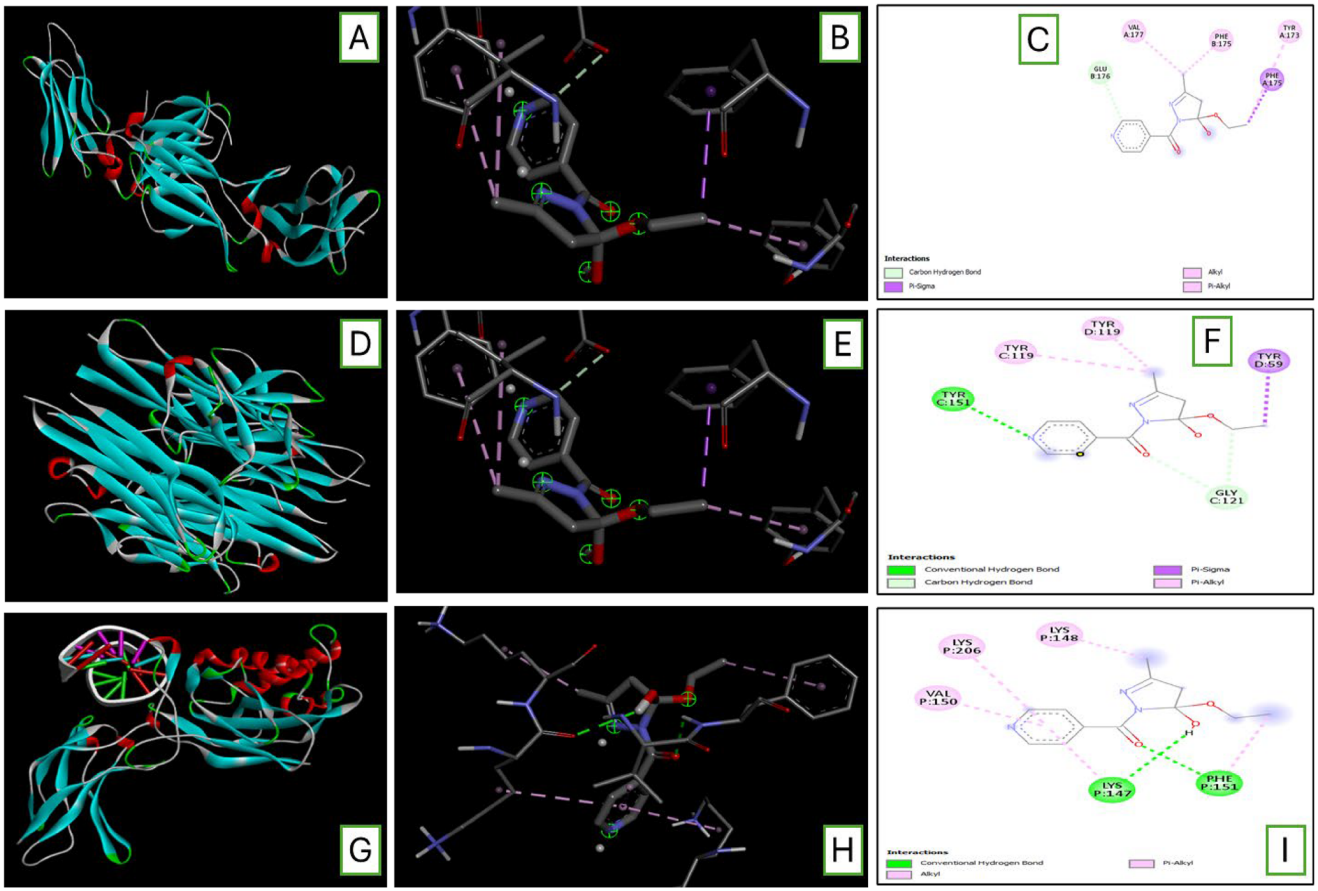
(A) 3D structure of IL‐10 spike receptor‐binding domain with PDB ID: 3LQM. (B) Surface area interactions of 5‐E‐5‐H‐PD with the receptor binding domain of IL‐10. (C) 2D interaction of (5‐E‐5‐H‐PD) and the receptor. (D) 3D structure of TNF‐α spike receptor‐binding domain with PDB ID: 2az5. (E) Surface area interactions of 5‐E‐5‐H‐PD with the receptor binding domain of TNF‐α. (F) 2D interaction of 5‐E‐5‐H‐PD and receptor protein. (G) 3D structure of TNF‐κB spike receptor‐binding domain with PDB ID: 1svc. (H) Surface area interactions of 5‐E‐5‐H‐PD with the receptor binding domain of NF‐κB. (I) A2D interaction of 5‐E‐5‐H‐PD and receptor protein.

Conformational changes observed due to the binding of 5‐E‐5‐H‐PD with IL‐10: PDB ID: 3LQM, as shown in Figure 6A–C, with TNF‐α: PDB ID: 2az5, as shown in Figure 6D–F, and with NF‐κB: PDB ID: 1svc, as shown in Figure 6G–I.

## 4 Discussion

The current work investigated the antiarthritic potential of a pyrazoline derivative in the CFA‐induced arthritis rat model, which demonstrated its significant therapeutic efficacy. The test compound showed a significant reduction in paw edema, arthritis scores, and serum pro‐inflammatory cytokines in a dose‐dependent manner. These results indicate the effectiveness of the pyrazoline derivative, 5‐E‐5‐H‐PD, in the treatment of inflammation and joint destruction associated with rheumatoid arthritis (RA).

According to numerous accounts, arthritis is thought to arise from an autoimmune mechanism in cases of localised hypoxemia. Arthritis pain may be primarily caused by increased oxygen demand and decreased blood flow in the area of damaged tissue [21]. Mostly synthetic therapeutic moieties such as glucocorticoids, nonsteroidal anti‐inflammatory medicines (NSAIDs), disease‐modifying anti‐rheumatic drugs (DMARDs), and some biologicals treat symptomatically rather than the underlying cause of the problem [22]. Furthermore, several surgical procedures might have serious adverse effects, such as post‐operative problems and psychological rejection [23]. Compared to patients on conventional medications, several studies revealed that recipients of these therapeutic moieties are more likely to suffer skin sensitivity and soft tissue sensitivity, infections in the genitourinary system, and weakness of bones and joints. As a result, it has been proposed that no drug has been able to effectively mitigate the effects of RA up to this point. Therefore, the development of novel drugs that can get past these obstacles will be highly sought after in the fight against RA [24].

According to the numerous studies, CFA can mimic a polyarthritis‐related inflammatory model in animals. Inflammation caused by CFA is initiated by the production of local chemical mediators like prostanoids and cytokines. If this acute phase persists, chemicals penetrate the inflammation site and cause chronic inflammation with the characteristics of hyperplasia, discomfort and swelling [25].

Although several plants and their derived natural compounds potentially treat inflammation in both in vivo and in vitro models of inflammation [26, 27, 28], recent studies have shown that pyrazoline derivatives have an anti‐arthritic effect because they have proven their potent anti‐inflammatory effect [8, 29, 30]. In the present study, the pyrazoline derivative, 5‐E‐5‐H‐PD, showed a protective effect against RA as evidenced by reduction of paw volume (Figure 2), arthritic scores (Figure 3), and improvement of histopathology of joint cartilage via reduction of inflammation and oxidative stress (Figure 5).

These findings are consistent with previous studies indicating that pyrazoline scaffolds possess anti‐inflammatory and analgesic properties due to their ability to inhibit cyclooxygenase enzymes and reduce prostaglandin synthesis. The high‐dose treatment group's reduction in TNF‐α, IL‐1β and IL‐6 levels aligns with literature suggesting that suppression of these cytokines plays a critical role in mitigating synovial inflammation and preventing joint damage in RA [31].

High levels of RF, CRP and anti‐CCP are a valid sign of the existence of inflammation, indicative of a higher probability of severe joint damage [32]. These are predictive surrogate markers for patients impeding the disease progression [33]. Studies have shown that in the untreated arthritic group, CRP and RF levels were significantly higher [1]. Our results are also consistent with the previous research showing elevated levels (Table 1). Liver enzymes were also elevated in arthritis patients [34]. In our study, the pyrazoline derivative, 5‐E‐5‐H‐PD, has not only lowered the levels of RF, CRP and anti‐CCP, but also decreased AST and ALT levels, thereby showing its potent anti‐arthritic potential as reported previously [35].

Haematological marker changes have been observed in RA patients. Similar alterations were seen in rats with CFA‐induced arthritis. In the disease group, WBC and ESR levels were enhanced, while Hb and RBC levels were decreased, which is why animals were suffering from anaemia. As the disease progresses, premature RBCs are destroyed quickly, which decreases erythropoietin and haemoglobin. WBC and platelet counts rise in the presence of disease or infection due to the activation of a complementary cascade of the immune system and activation of inflammatory modulators after the attack of an antigen [36]. In our study, the RA group has also shown the same haematological changes (Table 2). Numerous studies have established the connection between leucocytes and platelets in RA. To promote platelet transportation to the joint area, platelets cling to leukocytes in the bloodstream and boost leukocyte deposition on the endothelial wall [37]. Our compound has improved the haematological profile at all tested doses.

The primary pro‐inflammatory cytokines in RA are TNF‐α and IL‐6, which bind to receptors and cause bone injury. Thus, inhibiting them prevents/slows the deterioration of bone in RA‐positive patients [38]. The conventional inflammatory pathways known as NF‐κB signalling can stimulate osteoclast proliferation and differentiation, leading to erosion and bone destruction. So, it's a crucial target during the treatment of RA and other chronic immune‐mediated inflammatory illnesses. Inactivated NF‐κB is activated in the cytoplasm; inflammatory events cause it to be released and translocate to the nucleus [39, 40]. Table 4 displays the considerable rise in TNF‐α, IL‐10 and NF‐κB levels seen in the disease group due to CFA. The administration of 5‐E‐5‐H‐PD, a pyrazoline derivative, dramatically reduced the levels of inflammatory mediators in our investigation, indicating an anti‐inflammatory impact that may have been achieved by blocking signalling pathways (Table 4). Previous research indicates that synthetic pyrazoline derivative compounds can suppress TNFα expression and have strong anti‐inflammatory properties [41, 42].

Histopathological evaluation further supported the biochemical and clinical outcomes, revealing preservation of joint architecture, reduced synovial hyperplasia, and decreased infiltration of inflammatory cells in treated animals. This suggests that the pyrazoline derivative may exert both systemic anti‐inflammatory effects and localised joint protection.

One of the elements contributing to the pathophysiology of RA is oxidative stress. ROS are produced at higher levels in the synovium due to the inflammatory and hypoxic circumstances that are formed as the disease progresses. The severity of synovitis can also worsen due to increased ROS production [43]. ROS accelerate the severity of RA by causing mitochondrial damage and upregulating several inflammatory pathways, which in turn encourage bone deterioration [44]. Research has indicated that CFA injections and MDA production can be increased while SOD, GSH and CAT levels are suppressed [45]. Our results are also consistent with the research, as shown in Table 3. Studies have shown that pyrazoline derivatives hold promising potential as antioxidants [46], and our study has also shown antioxidant activity of 5‐E‐5‐H‐PD, a pyrazoline derivative, by restoring the changes induced by CFA (Table 3).

Molecular docking has been used recently to forecast the best positions for interactions between proteins and ligands. These studies are helpful in the recognition of the molecular structure of compounds and are useful for medicinal chemistry and drug discovery. By these methods, the most beneficial binding site for the ligand can be used to anticipate the chemical interactions that are indicated. In the current work, we attempted to examine the connection between IL‐10, TNF‐α, NF‐κB and 5‐E‐5‐H‐PD. In addition to pharmacologic evidence supporting its anti‐arthritic activity, 5‐E‐5‐H‐PD showed substantial binding affinities with each of these chosen targets (Figure 6 and Table 5A–C).

## 5 Conclusion

The findings of this study demonstrate the promising anti‐arthritic potential of the 5‐ethoxy 5‐hydroxy 3‐methyls 4, 5‐dihydro 1Hpyrazol 1 yl (pyridine 4 yl) methanone (5‐E‐5‐H‐PD) in the attenuation of the inflammation and joint damage of the Complete Freund's Adjuvant induced arthritis model. The current work showed significant improvement in the clinical scores, histopathological features, and pro‐inflammatory biomarker levels, which suggests its therapeutic efficacy. These findings further support the investigation into the test compound's mechanism of action and its potential development as a novel therapeutic moiety for rheumatoid arthritis. However, clinical translation requires extensive preclinical validation and safety profiling.

### 5.1 Limitations

First, the precise molecular mechanisms involved in the anti‐arthritic action were not fully elucidated. Second, the sample size was relatively small, and only two doses were tested. Lastly, long‐term toxicity, pharmacokinetics and comparative efficacy with existing DMARDs were not assessed, which are essential for clinical translation. Despite these limitations, the study provides compelling evidence that the tested pyrazoline derivative holds promise as a lead compound for RA treatment. Future studies should focus on mechanistic investigations, chronic models of arthritis, and formulation development to enhance bioavailability and targeted delivery.

## Author Contributions

Jian Li, Irfan Anjum, Faiza Naseer and Halima Qadir: conceptualization, supervision, data curation, writing the original draft, reviewing of the original draft. Mehreen Arif, Muhammad Riaz, Rabia Gul, Madiha Kanwal and Bei Zhuang: formal analysis, resources and software availability. Bei Zhuang: funding, writing and review of the original draft.

## Funding

The authors have nothing to report.

## Ethics Statement

The ethical approval for the conduction of the study was granted by the Animal Ethics Committee of Shifa College of Pharmaceutical Sciences, Shifa Tameer e Millat University (SCPS‐STMU/Animal Ethics Approval/IRB # 041‐24).

## Consent

The authors have nothing to report.

## Conflicts of Interest

The authors declare no conflicts of interest.

## Data Availability

The data that support the findings of this study are available on request from the corresponding author. The data are not publicly available due to privacy or ethical restrictions.
